# Functional connectivity and structural covariance between regions of interest can be measured more accurately using multivariate distance correlation

**DOI:** 10.1016/j.neuroimage.2016.04.047

**Published:** 2016-07-15

**Authors:** Linda Geerligs, Cam-CAN, Richard N. Henson

**Affiliations:** a MRC Cognition and Brain Sciences Unit, 15 Chaucer Road, Cambridge CB2 7EF, UK; b Cambridge Centre for Ageing and Neuroscience (Cam-CAN), University of Cambridge and MRC Cognition and Brain Sciences Unit, Cambridge, UK, http://www.cam-can.com

**Keywords:** Structural covariance, Functional connectivity, Multivariate, Distance correlation, Resting state, Graph theory

## Abstract

Studies of brain-wide functional connectivity or structural covariance typically use measures like the Pearson correlation coefficient, applied to data that have been averaged across voxels within regions of interest (ROIs). However, averaging across voxels may result in biased connectivity estimates when there is inhomogeneity within those ROIs, e.g., sub-regions that exhibit different patterns of functional connectivity or structural covariance. Here, we propose a new measure based on “distance correlation”; a test of multivariate dependence of high dimensional vectors, which allows for both linear and non-linear dependencies. We used simulations to show how distance correlation out-performs Pearson correlation in the face of inhomogeneous ROIs. To evaluate this new measure on real data, we use resting-state fMRI scans and T1 structural scans from 2 sessions on each of 214 participants from the Cambridge Centre for Ageing & Neuroscience (Cam-CAN) project. Pearson correlation and distance correlation showed similar average connectivity patterns, for both functional connectivity and structural covariance. Nevertheless, distance correlation was shown to be 1) more reliable across sessions, 2) more similar across participants, and 3) more robust to different sets of ROIs. Moreover, we found that the similarity between functional connectivity and structural covariance estimates was higher for distance correlation compared to Pearson correlation. We also explored the relative effects of different preprocessing options and motion artefacts on functional connectivity. Because distance correlation is easy to implement and fast to compute, it is a promising alternative to Pearson correlations for investigating ROI-based brain-wide connectivity patterns, for functional as well as structural data.

## Highlights

• We introduce distance correlation as a new measure of ROI-based connectivity.
• It can be used for functional connectivity and structural covariance analyses.
• Simulations show that distance correlation copes with inhomogeneous ROIs.
• In real data, distance correlation is more reliable and robust than Pearson correlation.
• Distance correlation improves correspondence between different ROI definitions.

## 1 Introduction

The brain is a network of a large number of regions, which may support different (cognitive) processes, but nonetheless interact with each other. In recent years, there has been much interest in the properties of this network, such as its modular structure and the existence of hub regions that help integrate information across brain regions (Bullmore and Bassett, 2011, Sporns and Betzel, 2016, Sporns et al., 2007). Such network analyses have become an important tool to characterize individual differences related to cognitive function, age and mental health (e.g. Alexander-Bloch et al., 2010, Brier et al., 2014, Crossley et al., 2014, Geerligs et al., 2014, Spreng and Turner, 2013, van den Heuvel et al., 2009). Three main, complementary techniques have been used to examine the network structure obtained from magnetic resonance imaging (MRI) of human participants. The first is diffusion-weighted MRI, which can be used to estimate the integrity of white-matter tracts between regions of interest (ROIs), but which is not considered here. Second is functional MRI (fMRI), in which connectivity within an individual is typically inferred by the correlation between time series of neuronal activity in each ROI. Third is structural MRI, from which the covariance between ROIs of a tissue property like grey matter volume or thickness can be examined across participants, which may reflect synchronized maturational changes in anatomically connected brain regions (Mechelli, 2005). In the remainder of this manuscript we will refer to these structural covariance analyses as estimates of structural connectivity.

MRI images typically contain of the order of 100,000 voxels, and there are several different parcellation schemes by which those voxels are grouped into ROIs. Some of these parcellations are adaptive, based on the data being analysed (Smith et al., 2013), but others typically come from a priori definitions, based on neuroanatomy (e.g. Tzourio-Mazoyer et al., 2002), task-based functional activations (e.g. Power et al., 2011) or prior functional connectivity results (e.g. Craddock et al., 2012, Gordon et al., 2014). Different studies use different parcellations, and ROIs selected on one criterion (e.g., neuroanatomy) may not respect divisions according to another criterion (e.g., functional activity). Once ROIs are defined, the relevant property of each ROI is normally reduced to a univariate measure by averaging the properties of voxels within that ROI (or by taking the first singular vector across voxels). Typically, the strength of connections between ROIs is then measured by the normalized covariance (Pearson correlation) across multiple measurements (time points or participants). Other methods have also been used, such as mutual information and time-lagged measures such as Granger causality, but these do not perform as well on typical fMRI data (Smith et al., 2011).

There are two distinct limitations to the ROI-based approach. First, important information might be lost by reducing each ROI to one dimension, given that techniques such as multi-voxel pattern analysis (MVPA) and representation similarity analysis (RSA) have demonstrated the importance of taking into account relative patterns across voxels (Kriegeskorte et al., 2008, Norman et al., 2006). This is especially likely for large ROIs, which are more likely to encompass distinct functional sub-regions (Gordon et al., 2014, Park et al., 2013). This problem is compounded when the same ROIs are used across participants, yet those participants have different functional organization, and/or there are errors in the coregistration of brain regions across participants. The second limitation is that covariance-based measures are not able to capture non-linear interactions between regions, yet previous studies have shown that shown that non-linear behaviour exists in regional interactions (Hlinka et al., 2011, Lahaye et al., 2003, Xie et al., 2008).

Here, we propose to use a different metric of connectivity that overcomes some of these limitations (though ultimately there is no substitute for good ROI definition). This metric is “distance correlation” (Székely et al., 2007), which estimates the multivariate dependence between high dimensional vectors, allowing for both linear and non-linear dependencies. Distance correlation therefore does not require reducing ROIs to one dimension, e.g., by averaging. We start with simulations showing how distance correlation out-performs Pearson correlation in the face of inhomogeneous ROI, and how it behaves according to ROI size, noise levels and temporal autocorrelation. We then apply distance correlation to real data, calculating ROI-by-ROI connectivity matrices for both functional and structural connectivity, and compared them with matrices obtained using the more standard Pearson correlation. More specifically, for functional connectivity, we compared the i) reliability across two scanning visits per participant, ii) similarity across a large number of individuals, iii) robustness to different sets of ROIs, and iv) robustness to different types of preprocessing and to effects of motion. For structural connectivity, we also compared reliability across two scanning visits, and furthermore, we compared the similarity of structural connectivity matrices with functional connectivity matrices.

## 2 Materials and methods

### 2.1 Participants

A sample of 236 participants (18–88 years old, M = 53.8, SD = 17.8, 119 males and 117 females) were taken from Stage 3 of the population-based sample of the Cambridge Centre for Ageing and Neuroscience (CamCAN). Participants were included if no brain abnormalities were detected, and if they completed both (f)MRI testing sessions. Participants had no contraindications to MRI, were native English-speakers, had normal or corrected-to-normal vision and hearing, scored 25 or higher on the mini mental state exam (MMSE; Folstein et al., 1975) and had no neurological disorders (see Shafto et al., 2014, for further details). Ethical approval for the study was obtained from the Cambridgeshire 2 (now East of England - Cambridge Central) Research Ethics Committee. Participants gave written informed consent.

### 2.2 fMRI data and image acquisition

Eyes-closed resting state functional magnetic resonance imaging (fMRI) data were collected in two separate scanning sessions, which were between three months and three years apart. MR data were collected as part of more extensive scanning sessions in a 3 T Siemens TIM Trio, with a 32 channel head-coil. The first scan lasted 8 min and 40 s (261 volumes) and the second scan lasted 5 min (152 volumes). Each volume contained 32 axial slices (acquired in descending order), with slice thickness of 3.7 mm and interslice gap of 20% (for whole brain coverage including cerebellum; TR = 1970 ms; TE = 30 ms; flip angle = 78 degrees; FOV = 192 mm × 192 mm; voxel-size = 3 mm × 3 mm × 4.44 mm). A high-resolution (1 mm × 1 mm × 1 mm) T1-weighted Magnetization Prepared RApid Gradient Echo (MPRAGE) image was acquired in both sessions. In the first session, we additionally acquired a T2-weighted structural image (1 mm × 1 mm × 1 mm) using a Sampling Perfection with Application optimized Contrasts using different flip angle Evolution (SPACE) sequence.

### 2.3 Data pre-processing

Pre-processing was performed using the SPM12 software (http://www.fil.ion.ucl.ac.uk/spm), as called by the automatic analysis (AA) batching system (http://imaging.mrc-cbu.cam.ac.uk/imaging/AA). For full details, see Taylor et al. (in press). In brief, fieldmaps were used to undistort the functional EPI images, which were then motion-corrected and slice-time corrected. For the first session, the T1 and T2 images were combined in order to segment various tissue classes using unified segmentation, including grey matter (GM), white matter (WM) and cerebrospinal fluid (CSF). For the second session, only the T1 images were used for segmentation. The GM and WM segments for each participant were used to create a sample-specific anatomical template, using the DARTEL procedure to optimize inter-participant alignment, separately for each session. The template for each session was subsequently transformed into MNI space, using a 12-parameter affine mapping. The EPI images were then coregistered to the T1 image, and the DARTEL flowfields and MNI transformation applied to the EPI images. The segmented images were also used to create WM and cerebrospinal fluid (CSF) masks for each participant by selecting only voxels with less than 1% of grey matter and more than 80% of WM/CSF. For the EPI images and the WM and CSF segments, we applied the DARTEL deformations and MNI transformation to the original images; for the structural connectivity analysis, we applied an addition modulation step (modulating by the Jacobean of the deformations) in order to preserve the amount of signal in the images (similar to voxel-based morphometry analyses; Ashburner and Friston, 2000).

### 2.4 Extended pre-processing and ROI extraction

To reduce the effects of motion on the functional connectivity results, we used a combination of approaches. The first of these was to apply the Wavelet Despike method for removing motion artefacts from fMRI data without the need for data scrubbing (Patel et al., 2014). The method detects irregular events at different frequencies by identifying chains of outlying wavelet coefficients, and projects these out of the voxel time series. The algorithm can remove both prolonged motion artefacts, such as spin-history effects, as well as higher frequency events such as spikes. The total amount of despiking performed on a dataset is quantified by the percentage of voxels containing a spike in that volume of data. Participants with an average spike percentage, in any of the mental states, of two standard deviations above the mean across all mental states (6.55%), were excluded from further analysis. This led to the exclusion of 19 participants. Four additional participants were excluded due to normalization problems, leaving a total of 214 participants included in the analyses. To quantify the total motion for each participant, the root mean square volume-to-volume displacement was computed using the approach of Jenkinson et al. (2002).

Data were extracted for each voxel in three different sets of ROIs; 264 regions defined by Power et al. (2011); 116 regions in the Automated Anatomical Labelling (AAL) atlas (Tzourio-Mazoyer et al., 2002); and 748 of the 840 regions defined by Craddock et al. (2012). Only the 748 regions were included that had sufficient coverage in our recent paper using a superset of the participants included here (Geerligs et al., 2015), which allowed us to use existing network labels defined in our previous study. For each ROI set, we excluded ROIs with insufficient coverage in the functional images from either session for any of the participants (less than 25% overlap with mask of functional images at 70% mean signal intensity). Voxels outside this mask were not used in the functional connectivity analyses. This led the exclusion of 2 Craddock ROIs (746 remaining), 19 Power ROIs (245 remaining) and 2 AAL ROIs (114 remaining).

The second step to reduce the effects of motion and other noise confounds on functional connectivity results was to apply a general linear model (GLM). This model included expansions of the six original motion parameters, as well as of average signals in the WM and CSF from the time courses of each voxel within each ROI. The WM and CSF signals were created by averaging across voxels in the associated mask image, after the Wavelet despiking. The expansions included the first-order temporal derivative, as well as their squares and squared derivatives, which recent research has shown reduces the effects of motion (Satterthwaite et al., 2013). In total, there were 32 confound and noise regressors. A high-pass filter (0.008 Hz), or band-pass filter (0.008–0.1 Hz) was implemented by including a discrete cosine transform (DCT) set in the GLM. Unless mentioned otherwise, analyses reported in the results section are based on high-pass filtered data. The autocorrelation in the GLM residuals was modelled by a family of 8 exponentials with half-lives from 0.5 to 64 TRs, given evidence that an AR(1) plus white noise model is not sufficient for resting-state data (Eklund et al., 2012). The autocorrelation hyperparameters were estimated from pooling across voxels within each ROI, using Restricted Maximum Likelihood Estimation (ReML), but separately for each ROI, in order to account for differences in autocorrelation between cortical and subcortical regions (see Section 3.3). The autocorrelation model was inverted in order to prewhiten the data (Friston et al., 2002) and functional connectivity was then estimated from the whitened residuals of this model.

For structural connectivity, we extracted the grey matter volumes for each voxel within each ROI in the three ROI sets. Across participants, we regressed out differences that were associated with total grey matter volume, as we were specifically interested in regional variability (Peelle and Cusack, 2012). The residuals of this regression were used in the structural connectivity analyses below.

### 2.5 Connectivity measures

Three measures of connectivity were computed for each pair of ROIs in the three sets; Pearson correlation, univariate distance correlation and multivariate distance correlation. The Pearson correlation and the univariate distance correlation were computed after averaging the signals across all voxels within each ROI. The multivariate distance correlation was computed based on all the voxels in each of the two ROIs.

Pearson correlation is a measure of the linear dependence between two signals. For two time series, *x* and *y*, with *n* time points, the Pearson correlation is given by:

$$\mathrm{Pco}r_{\mathrm{xy}}=\frac{\sum_{i=1}^{n}\left(x_{i}-\overset{̅}{x}\right)\left(y_{i}-\overset{̅}{y}\right)}{\sqrt{\sum_{i=1}^{n}{\left(x_{i}-\overset{̅}{x}\right)}^{2}}\sqrt{\sum_{i=1}^{n}{\left(y_{i}-\overset{̅}{y}\right)}^{2}}}$$

where $\overset{̅}{x}$ is the mean of *x* and $\overset{̅}{y}$ is the mean of *y*.

In contrast, distance correlation is a more general measure of dependence of two signals or two sets of signals, which can be either linear or non-linear (Székely et al., 2007). For the multivariate connectivity measure, we used the unbiased estimate of distance correlation, which is not biased by the number of voxels in a region (Székely and Rizzo, 2013). Let us define *X* and *Y* as two matrices of *n* time points by *v* voxels. Prior to distance correlation, each voxel's time series should be variance normalized (z-scored). The first step in computing distance correlation is to compute the Euclidean distance in voxel-space between each pair of time points for *X* and *Y* separately:

$$a_{i,j}=\sqrt{\sum_{k=1}^{v}{\left(X_{\mathrm{ik}}-X_{\mathrm{jk}}\right)}^{2}}\;i,j=1,\ldots .,n,$$

$$b_{i,j}=\sqrt{\sum_{k=1}^{v}{\left(Y_{\mathrm{ik}}-Y_{\mathrm{jk}}\right)}^{2}}\;i,j=1,\ldots .,n.$$

For the univariate distance correlation, we apply double-centering to the Euclidean distance matrices *a* and *b*:

$$A_{i,j}=a_{i,j}-{\overset{̅}{a}}_{i.}-{\overset{̅}{a}}_{.j}+{\overset{̅}{a}}_{..},$$

where ${\overset{̅}{a}}_{i.}$ is the *i-*th row mean, $\overset{̅}{a}._{j}$ is the *j*-th column mean and ${\overset{̅}{a}}_{..}$ is the grand mean of the distance matrix of X (and likewise for the centred matrix *B* derived from *b*).

For the multivariate distance correlation, we apply U-centering instead of double-centering, in order to ensure that the correlation estimates are not biased by the number of voxels in an ROI (Székely and Rizzo, 2013, Szekely and Rizzo, 2014). U-centering ensures that row and column means are zero and that all expected values are zero.

$$A_{i,j}=\left\{\begin{array}{ll} a_{i,j}-\frac{1}{n-2}\sum_{l=1}^{n}a_{i,l}-\frac{1}{n-2}\sum_{k=1}^{n}a_{k,j}+\frac{1}{\left(n-1\right)\left(n-2\right)}\sum_{k,l=1}^{n}a_{k,l}, & i\neq j;\\ 0, & i=j. \end{array}\right)$$

These centred distance matrices are then used to compute the distance covariance and distance variance:

$$\mathrm{dCo}v\left(X,Y\right)=\frac{1}{K}\sum_{i,j=1}^{n}A_{i,j}B_{i,j}$$

$$\mathrm{dVa}r\left(X\right)=\frac{1}{K}\sum_{i,j=1}^{n}A_{i,j}^{2}.$$

For the double-centred version, the normalization factor *K* = *n*^2^, whereas for the U-centred version, *K* = *n*(*n* − 3).

Finally, distance correlation is defined as:

$$\mathrm{dCor}\left(X,Y\right)=\left\{\begin{array}{c} \sqrt{\frac{\mathrm{dCov}\left(X,Y\right)}{\sqrt{\mathrm{dVar}\left(X\right)\;\text{dVar}\left(Y\right)}}}\;\mathrm{dCo}v\left(X,Y\right)>0\\ 0,\;\mathrm{dCo}v\left(X,Y\right)\leq 0 \end{array}\right).$$

Because Dcor is a measure of the similarity of distances between time-points (rather than distances from the mean in the case of Pcor), it is not possible to distinguish between negative and positive associations between regions. The Euclidean distance matrices of two perfectly negatively correlated signals (*a* and *b*, with the same mean and SD) would be as similar to each other as the distance matrices of two perfectly positively correlated signals. Dcor is therefore generally positive, though it is possible to get negative estimates for the U-centred (unbiased) estimate, because the distribution of Dcor under the null-hypothesis is shifted the left. Such negative estimates of Dcor (after U-centering) imply that the dependence between two signals is non-significant. Indeed, connections that had a negative Dcor typically also had a non-significant Pcor. This was true for 97% of the zero connections for the Craddock ROIs, 99% for the Power ROIs and 100% for the AAL ROIs (in the structural connectivity analyses). We therefore set cases of negative Dcor to zero. For the functional connectivity data, this occurred for 0.02% of connections for the Craddock ROIs and 0.1% for the Power ROIs, while it did not occur for the AAL ROIs. For structural connectivity, this occurred for 28% of connections for Craddock ROIs, 34% for Power ROIs and 0.3% for AAL ROIs. Note that, for univariate data with a bivariate normal distribution, Dcor can be derived from the absolute value of Pcor (Székely et al., 2007). Matlab scripts for computing the double-centred and the U-centred estimates of Dcor are available from http://imaging.mrc-cbu.cam.ac.uk/imaging/Geerligs_DistCor. We also provide an example script for functional connectivity analyses, which includes the extraction of data from ROIs, pre-whitening and nuisance regression. The two versions of Dcor have also been implemented by Rizzo and Székely in the *energy* package for R (Rizzo and Szekely, 2014).

### 2.6 Simulations

We start with some simulations to illustrate differences between Pcor and Dcor, and to explore potential causes of bias in the Dcor estimates. We simulated two ROIs, consisting of either one or two sets of voxels with distinct time series (M = 0, SD = 1) generated from a multivariate normal distribution. Each voxel expressed one of the time series, plus a small amount (SD = 0.2) of independent, random noise.

We first simulated cases in which multivariate connectivity produces results that differ from a univariate measure. We simulated four different signals, two of which were present in ROI 1 and the other two in ROI 2. In the first case, the two signals from the same ROI were negatively correlated (r = − 1), while each of these signals correlated positively (r = 0.5) with one of the signals in the other ROI (each signal was present in 10 of the 20 voxels). In the second case, we simulated a situation where both ROIs contained one signal, which was only present in half of the voxels. The other voxels contained an uncorrelated signal, which was different in the two sets of ROIs.

We also investigated a number of situations where connectivity estimates may be biased. In each case, each ROI contained only one distinct signal, which were either correlated (r = 0.5) or uncorrelated (r = 0) across ROIs. First, we investigated the effects of varying the number of voxels (20 or 40). Second, we investigated the effects of noise by increasing the voxel-specific noise (SD = 2), or adding ROI-specific noise with either the same (SD = 0.55) or different variance in the two ROIs (SD = 0.2 and SD = 0.8). Third, we simulated the effect of autocorrelation in the signals, using the same procedure as Arbabshirani et al. (2014). In this case, the signals in the two ROIs were based on two signals *w* and *z*, which were generated from a multivariate normal distribution (r = 0 or r = 0.5, as above). The autocorrelation in these signals was adapted according to:

$$x_{t}=\alpha x_{t-1}+w_{t}$$

$$y_{t}=\beta y_{t-1}+z_{t}$$

where *α* and *β* varied independently (and *x*_1_ = *w*_1_ and *y*_1_ = *z*_1_).

### 2.7 Evaluation of the connectivity measures

We used different approaches to compare the different estimates of functional and structural connectivity. All analyses were performed on data from session 1, except from analyses of reliability of connectivity estimates.

The first was to compute the intra-class correlation (ICC) between the (vectorized) connectivity matrix for session 1 and the connectivity matrix for session 2 for each participant. In this way, we tested the reliability of the network structure. We also performed this analysis for regional connectivity patterns, where the vector of connections between one ROI and all other ROIs was correlated between different sessions. Paired samples t-tests were used to determine if there were significant differences between Pcor and Dcor in these reliability estimates.

For structural connectivity we only had one connectivity matrix across participants, so we computed the Spearman correlation between the connectivity matrices in session 1 and session 2. To test whether this reliability estimate was significantly different between Pcor and Dcor estimations of connectivity, we used permutation tests. We randomly swapped Pcor and Dcor estimates for the same connections (across both sessions), and recomputed the correlation difference. The p-value was computed as the fraction of cases in which this null-distribution (of 5000 permutations) resulted in a larger (absolute) difference than the observed difference in the real data.

Second, we computed the ICC between the vector of all participants' connectivity estimates from session 1 and the vector of connectivity estimates for session 2, for each connection separately (only for the functional connectivity matrices).

Third, we assessed the similarity of the functional connectivity network structure between different participants by computing the Pearson correlation coefficient between each participant's connectivity matrix and the average connectivity matrix across participants.

Fourth, we assessed how the regional change in reliability between Pcor and Dcor was associated with the homogeneity of each ROI. For each ROI, we computed the whole-brain connectivity pattern of each voxel to all other ROIs, using Pearson correlations. For each participant, we entered the connectivity patterns from all voxels in an ROI into a principal component analysis (PCA). Note that PCA ignores the mean of each voxels' connectivity pattern, ensuring that the average connectivity strength to all other ROI did not directly affect our measure of homogeneity. The homogeneity of the parcel was calculated as the percent of total variance across all voxels' connectivity patterns that can be explained by the first principal component. Higher homogeneity indicates that the connectivity patterns of voxels within an ROI can be better described by a single pattern of connectivity.

Finally, we assessed the robustness of the functional connectivity measures to the choice of ROI set. Because ROI sets cannot be compared directly, we first projected the ROI-ROI connectivity matrices onto voxel-voxel connectivity matrices. Then we computed the correlation between voxel-wise connectivity matrices from different ROI sets, using only those voxels that were present in both sets of ROIs.

## 3 Results

### 3.1 Simulations — Advantages of a multivariate method

**Fig. 2:**
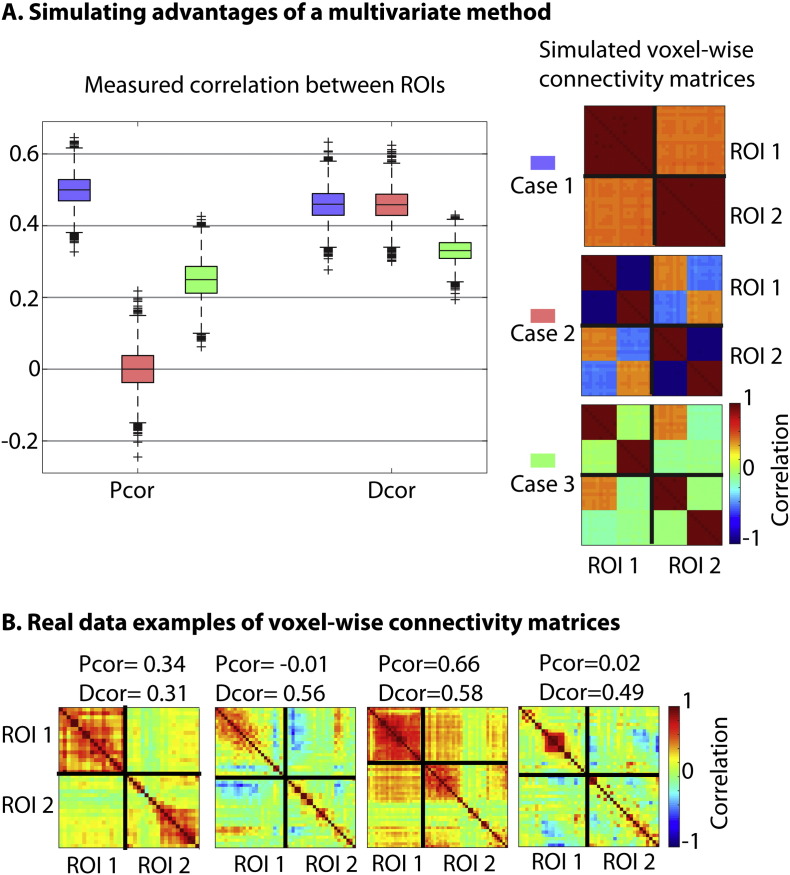
**A**. Demonstration of some important differences between the Pcor and Dcor methods. We simulated two ROIs with different voxel-wise connectivity patterns, which are shown in the figures on the right. The boxplots show the observed Dcor and Pcor estimates between the two ROIs in three different cases. **B**. Examples of voxel-wise connectivity patterns between two Craddock ROIs in real data.

We start with a number of simulations that illustrate differences between the Pearson correlation (Pcor) and distance correlation (Dcor). As a baseline, we simulated a case where connectivity was truly univariate, i.e., the same connectivity pattern was present across all voxels in two ROIs (Case 1 in Fig. 2). It should be noted that Dcor and Pcor are not directly comparable (Székely et al., 2007). For example, at r = 0.5 (the case simulated here), Dcor tends to be slightly lower than Pcor. However, we can compare the effect of other variables on each measure. For example, in the extreme case where each ROI consists of two equal-size subsets of voxels that are anticorrelated (Case 2 in Fig. 2), no significant connectivity can be detected with Pcor, while the multivariate connectivity measured with Dcor is the same as in the univariate (baseline) case. Case 3 is perhaps a more realistic example of this, where each ROI consists of two distinct subsets of voxels, only one subset of which is correlated across ROIs (r = 0.5). Here, we again see that the drop in connectivity estimates from the baseline case is larger for Pcor than Dcor. To demonstrate that these examples occur in reality, Fig. 2B shows examples from the Craddock ROIs in our real data that express situations similar to our simulations.

### 3.2 Simulations — potential causes of bias

**Fig. 3:**
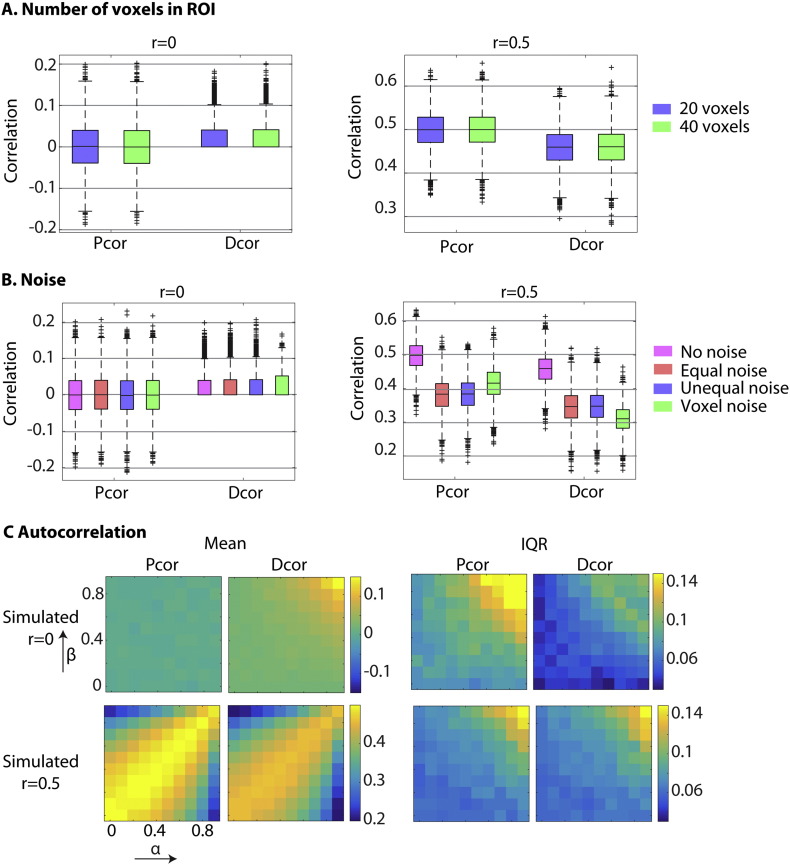
Simulations of potential sources of bias in the Dcor and Pcor connectivity estimates. **A**. Effects of varying the number of voxels in an ROI. **B**. Effects of varying the type and the amount of noise in each ROI. **C.** Effects of varying the autocorrelation of the signals in each ROI. The *α* and *β* parameters indicate the amount of autocorrelation in each ROI (see Section 2.6). IQR = inter-quartile range.

In Fig. 3, we show the result of simulations that explored potential biases in the Dcor estimates. We considered the two cases of when the true univariate correlation was either 0 or 0.5. First, note that when there is no true correlation, Dcor values approach 0, but because our Dcor estimate is unsigned (after removing occasional negative values caused by U-centering; see Materials and methods), the distribution of estimates is positively skewed above 0. This is why we used unsigned Pcor estimates (where the sign of negative correlations was flipped) when comparing reliability of Dcor and Pcor in real data later.

In order to compare Dcor values across connections, it is important that they are not biased by different numbers of voxels within different ROIs. Fig. 3A confirms that the Dcor estimates are not affected by ROI size, owing to our use of U-centering (see Materials and methods).

Next, we simulated the effects of noise (Fig. 3B). When the same noise was added to all voxels in an ROI (ROI-specific noise), Pcor and Dcor showed approximately equivalent declines in their connectivity estimates as the amount of this noise increased (when the true r = 0.5), regardless of whether or not the amount of that noise was equal across ROIs. However, when the noise was independent across voxels (voxel-specific noise), Dcor showed a larger decline than Pcor. This is because, with Pcor, the voxel-specific noise is attenuated by averaging across voxels within an ROI. The relative level of ROI-specific vs. voxel-specific noise in real data is considered in the next section. Nonetheless, this general decrease in estimated connectivity as noise increases is expected: more important is the finding that, when there was no true connectivity between ROIs, adding different types of noise did not bias the estimates of Dcor.

Finally, we simulated the effects of autocorrelation in the time-series (Fig. 3). Both Pcor and Dcor have the assumption that time-points should be exchangeable, which is violated when there is strong autocorrelation in the signal. We varied the autocorrelation in two ROIs separately, by varying the parameters of a first-order autoregressive model (*α* and *β* parameters Section 2.6). For both Dcor and Pcor, the variability (inter-quartile range, IQR) of connectivity estimates increased as the autocorrelation increased (right panels in Fig. 3C). This reinforces the problem of autocorrelation for correlational measures of connectivity (Arbabshirani et al., 2014). In the case of r = 0, this increased variability results in an overestimation bias in the case of Dcor (left panels of Fig. 3). Furthermore, in the case of r = 0.5, we found that both Dcor and Pcor produced an underestimation bias as the difference in autocorrelation across the two ROIs increased. These results emphasize the problem of auto-correlation for both Dcor and Pcor, which we turn to next, in the context of the high-/band-pass filtering normally applied to fMRI data.

### 3.3 Addressing potential sources of bias in real data

In our simulations, we found that two factors could potentially bias our connectivity estimates. The first of these was the negative bias in Dcor estimates associated with voxel-specific noise. High levels of such noise had a larger impact on Dcor than Pcor estimates. In reality, it is unlikely that such a high level of noise exists that is independent across voxels, given the typical point-spread function of BOLD contrast at 3 T (Parkes et al., 2005). Nonetheless, if voxel-specific noise was impacting our results, we would expect that ROIs with more dissimilar voxels (low correlation on average between the time-courses within an ROI) would have lower Dcor estimates. We investigated this for the set of Craddock ROIs by correlating the average connectivity between each ROI and all other ROIs, with the average correlation between the time series of all voxels within an ROI. We observed the opposite effect to what we would expect if Dcor were biased due to voxel-wise noise; for Pcor, regions with more dissimilar voxels had lower (absolute) connectivity estimates (r = 0.33, p < 0.001), while for Dcor, regions with more dissimilar voxels tended to have slightly higher connectivity estimates (r = − 0.14, p < 0.001). For structural connectivity, we found that regions with more dissimilar voxels tended to have lower connectivity estimates for both Dcor (r = 0.18, p < 0.001) and (absolute) Pcor values (r = 0.24, p < 0.001). Because these associations are stronger for Pcor than Dcor, for both functional and structural connectivity, these results suggest that voxel-specific noise did not result in a substantial or consistent bias of Dcor connectivity estimates in the present data.

The second potential bias arises from autocorrelation in the data, which can lead to a positive bias in Dcor when two regions are not connected, and can bias both Pcor and Dcor when the autocorrelation differs between regions. The degree of autocorrelation depends on any temporal filtering performed on the data, and many functional connectivity analyses either high-pass or band-pass filter their data, in order to remove various noise sources. After high-pass filtering to 0.008 Hz, our data showed significant lag-1 autocorrelation, which was stronger in the cortical (normalized lag-1 correlation, r1 = 0.16, averaged across participants and brain regions for the Craddock ROIs) than subcortical regions (r1 = 0.04). We therefore pre-whitened the data (see Materials and methods), which successfully removed any significant lag-1 autocorrelation in cortical (r1 < 0.001) or subcortical regions (r1 = − 0.03). This pre-whitening resulted in a significant improvement in the reliability of the functional connectivity matrix across scans for Pcor (see Fig. 5E; Power, Cohen's d = 0.19, t(213) = 2.7, p = 0.007; Craddock, d = 0.36, t(213) = 5.3, p < 0.001; AAL, d = 1.05, t(213) = 15.3, p < 0.001). We also observed that the between-participant similarity of Pcor improved after pre-whitening the data for the Craddock and AAL ROIs (Fig. 5F; Craddock, d = 0.45, t(213) = 6.6, p < 0.001; AAL, d = 1.05, t(213) = 15.4, p < 0.001), though not the Power ROIs (d = 0.01, t(213) = 0.21, p = 0.83).

After band-pass filtering from 0.008 Hz to 0.1 Hz, the lag-1 autocorrelation was even higher (r1 = 0.77), and could not be removed by our pre-whitening procedure (r1 = 0.75). In line with our simulations, we observed that the variability across participants for Pcor estimates was higher for the band-pass filtered data (with high autocorrelation; Power ROIs r = 0.19; Craddock r = 0.19, AAL r = 0.19), than for high-pass filtered data, either with (Power ROIs r = 0.14; Craddock r = 0.14, AAL r = 0.15) or without (Power ROIs r = 0.15; Craddock r = 0.16, AAL r = 0.17) pre-whitening. When we applied Dcor to band-pass filtered data, it was the weaker connections than tended to become stronger, confirming the positive bias we observed in our simulations. We also found that the reliability of both Pcor (Power, d = 1.92, t(213) = 28.1, p < 0.001; Craddock, d = 2.18, t(213) = 31.9, p < 0.001; AAL, d = 2.18, t(213) = 31.9, p < 0.001) and Dcor (Power, d = 4.39, t(213) = 64.2, p < 0.001; Craddock, d = 4.6, t(213) = 67.3, p < 0.001; AAL, d = 4.99, t(213) = 73.1, p < 0.001) were significantly lower after band-pass filtering as compared to high-pass filtering. These results suggest that it is not appropriate to use Dcor on band-pass filtered data, unless the autocorrelation can be fully accounted for.

### 3.4 Differences between the Pearson correlation and distance correlation connectivity estimates

**Fig. 4:**
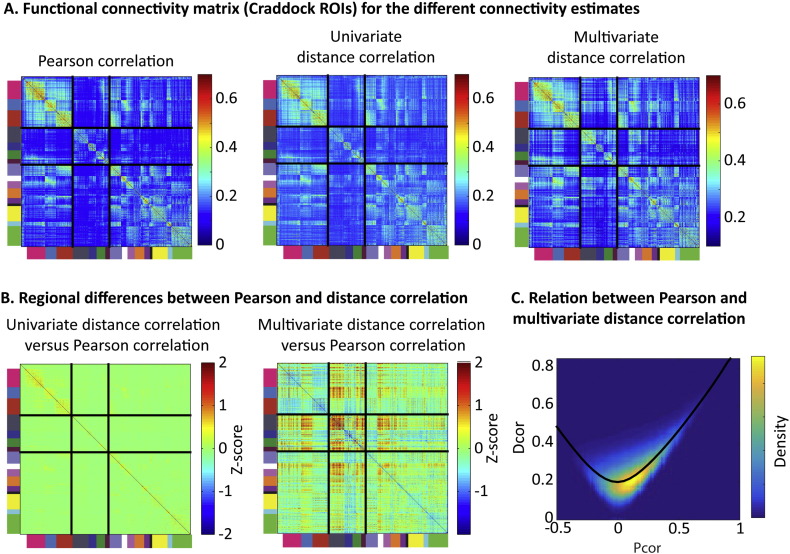
**A**: Functional connectivity matrices based on the set of Craddock ROIs for Pearson correlation (Pcor) and univariate and multivariate distance correlation (Dcor). Unsigned Pcor estimates were used to emphasize differences that were due to the multivariate method, rather than the absence of negative correlations. ROIs are ordered by functional network, as indicated by the colors on the left side and bottom of the functional connectivity matrices. **B**: Illustration of the differences between univariate Dcor and Pcor, and between multivariate Dcor and Pcor. For this illustration, we Z-transformed the connectivity matrices (based on mean and standard deviation over all elements of the matrix), and subtracted the z-scores of Pcor from Dcor. **C**. Density plot of the association between Pcor and Dcor for average functional connectivity across participants. The black line indicates the average Dcor estimates corresponding to each value of Pcor.

The connectivity matrices for univariate Pearson correlation (Pcor) and multivariate distance correlation (Dcor) for the set of Craddock ROIs are shown in Fig. 4A. Overall, the functional connectivity architecture was similar for both methods, with similar network structure apparent. To quantify the similarity between measures, we used unsigned Pcor estimates (i.e, flipped the sign of negative Pearson correlations), since Dcor cannot distinguish between negative and positive correlations. The correlation between the average connectivity matrices for both measures was r = 0.86 (the corresponding figures for the Power ROIs and AAL ROIs were 0.9 and 0.82). To illustrate this association between Pcor and Dcor values, we show a density plot in Fig. 4C.

**Fig. 5:**
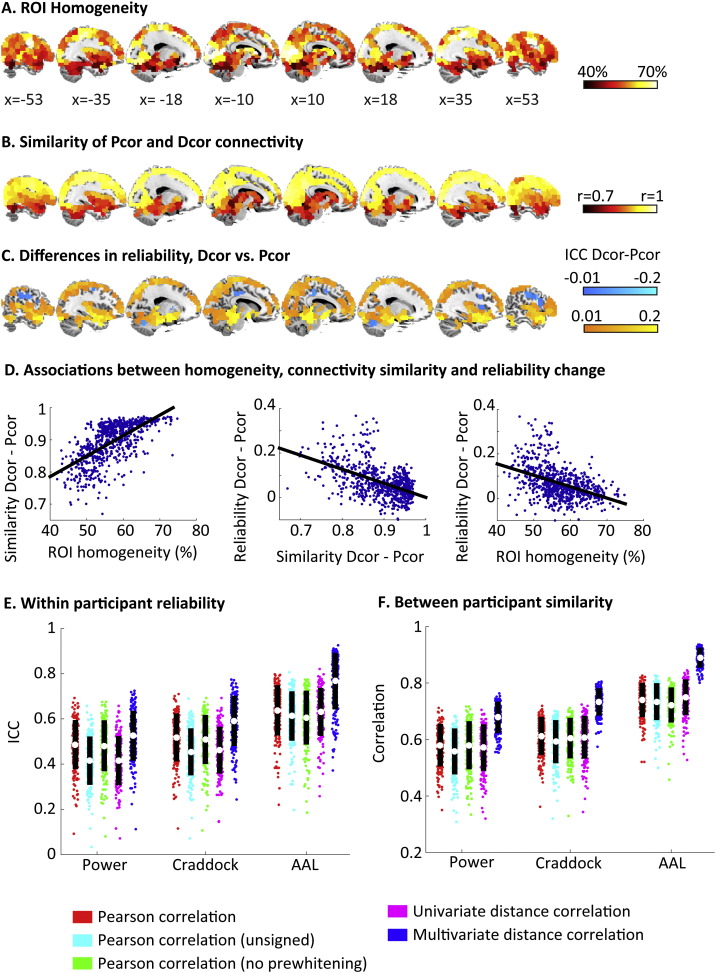
**A**: Regional differences in ROI homogeneity, as measured by the percentage of variance in its functional connectivity patterns that could be explained by the first principal component. **B**. Correlation between Pcor and Dcor functional connectivity patterns from each brain region to all other regions. Unsigned Pcor values were used to quantify the similarity. **C**: Differences between Dcor and Pcor in the reliability of regional connectivity patterns (ICC between session 1 and session 2). Red–yellow regions show significantly (p < 0.001) higher reliability for Dcor compared to Pcor; blue regions show significantly higher reliability for Pcor compared to Dcor. **D**. Associations between the measures of connectivity differences, reliability differences and ROI homogeneity shown in the panels A–C above. **E**. Scatterplots showing the mean and standard deviation of the within-participant reliability and **F**. between-participant similarity of the different functional connectivity measures for each of the three ROI sets.

The connectivity differences for the Craddock ROIs are highlighted in Fig. 4B. The most notable differences were found in connections of the brainstem and the thalamus, which were stronger for the multivariate connectivity measure. This was true for connections within the brainstem, connections between the thalamus and the brainstem, and connections from the thalamus and brainstem to the anterior insula and auditory network (see also Fig. 5). Next, we examined whether these differences in connectivity estimates were due to the non-linear nature, or the multivariate nature, of distance correlation. To this end, we computed a univariate version of Dcor, using the average signal, instead of the multivariate signal, within each ROI. The connectivity patterns we observed for univariate Dcor were nearly identical to the ones we observed with Pcor (r > 0.99 for all three ROI sets), suggesting that it is the multivariate nature of Dcor that leads to the observed differences in connectivity estimates, rather than non-linear dependencies.

The advantage of a multivariate over univariate Dcor suggests it is those ROIs that are not homogenous (e.g. contain voxels with dissimilar time courses) that should show connectivity patterns that differ between Pcor and multivariate Dcor. To examine this link, we first assessed the homogeneity of each ROI by examining how much of the variance of each voxel's connectivity pattern to all other ROIs could be explained by the first principal component from a principal component analysis. Next, we assessed regional differences in connectivity between Pcor and Dcor. This was done by correlating the vector of connectivity values (average connectivity values between one ROI and all other ROIs) between the two measures. We found a strong association between ROI homogeneity and the similarity between Pcor and Dcor connectivity patterns; ROIs in which a large proportion of the variance was captured by the first principal component showed similar connectivity patterns (r = 0.66, p < 0.001), as shown in Fig. 5A and B for the Craddock ROIs. A similar relation was observed for the AAL ROIs (r = 0.37, p < 0.001) and the Power ROIs (r = 0.68, p < 0.001). It was particularly the ROIs in subcortical regions, including the thalamus, basal ganglia, brainstem and cerebellum, that had relatively low homogeneity, and hence low similarity between Pcor and Dcor measures. On average, ROI homogeneity was highest for the Power ROIs (mean variance explained by one component = 62.1%), lower for the Craddock ROIs (M = 56.9%) and lowest for the AAL ROIs (M = 46.4%).

### 3.5 Within-participant reliability

Fig. 5E shows the reliability of the obtained network structure for the two connectivity measures by computing the ICC between the connectivity matrices in session 1 and session 2. We found that the reliability of the connectivity estimates increased markedly for Dcor compared to Pcor in each of the ROI sets (Power ROIs, d = 0.59, t(213) = 8.7, p < 0.001; Craddock ROIs, d = 0.95, t(213) = 13.8, p < 0.001 and AAL ROIs, d = 1, t(213) = 14.8, p < 0.001).

The regional patterns of reliability change in Dcor compared to Pcor are shown in Fig. 5C. ROIs with the largest improvement in reliability were also the ones that showed the biggest change in their connectivity patterns (Craddock, r = 0.52, p < 0.001; Power, r = 0.3, p < 0.001; AAL, r = 0.39, p < 0.001) and the ones with the lowest homogeneity (at least for Craddock, r = 0.44, p < 0.001, and Power, r = 0.2, p = 0.002, ROIs, though not for AAL ROIs, r = 0.09, p = 0.36). Improvements in reliability were found in multiple places over the brain, but most prominently in the insula, cerebellum and brainstem. Some regions showed poorer reliability for Dcor compared to Pcor, primarily in middle frontal/parietal regions, as well as some areas in the cingulate. This may be associated with the smaller range of Dcor values compared to Pcor values, as no significant decreases in reliability were observed when unsigned Pcor values were used in the analysis.

To test whether the increase in reliability was due to the multivariate nature of the test, we compared Pcor with univariate Dcor. For univariate Dcor, compared to Pcor, we observed worse reliability for the Power ROIs (d = − 1.23, t(213) = − 18, p < 0.001), the Craddock ROIs (d = − 1.05, t(213) = − 15.4, p < 0.001) and the AAL ROIs (d = − 0.17, t(213) = − 2.5, p = 0.01), again suggesting that it is the multivariate rather than nonlinear nature of Dcor that is important for its improved reliability.

To test how the difference in range of Dcor (0 to 1) versus Pcor (− 1 to 1) affected the reliability estimates, we also compared Dcor to unsigned Pcor values (0 to 1). The improvement in reliability for Dcor was even larger relative to these unsigned Pcor values (Power ROIs, d = 1.94, t(213) = 28.4, p < 0.001; Craddock ROIs, d = 1.97, t(213) = 28.8, p < 0.001 and AAL ROIs, d = 1.25, t(213) = 18.3, p < 0.001).

Some software (e.g. SPM) uses the first singular vector of the time-by-voxel data matrix to represent the time series of an ROI. In theory, this allows for some inhomogeneity in the ROI, by weighting the contributions of each voxel differently, rather than simply taking the (unweighted) mean across voxels. However, when we repeated the present Pcor analyses with the first singular vector, the reliability was significantly worse than taking the mean; for the Power ROIs (d = 0.54, t(213) = 7.9, p < 0.001), the Craddock ROIs (d = 2.78, t(213) = 40.6, p < 0.001) and the AAL ROIs (d = 2.73, t(213) = 40, p < 0.001). Thus, taking the first singular vector of each ROI is unable to accommodate ROI inhomogeneity in the same way that multivariate Dcor can.

### 3.6 Between-participant similarity

When ROIs are not perfectly homogeneous, different voxels may dominate the ROIs average signal for different participants. This could result in exaggerated differences between participants when Pcor is used (over and above any differences in within-participant reliability). Therefore, we compared the similarity of connectivity patterns between participants for Pcor and multivariate Dcor. We observed a substantial increase in the similarity of connectivity matrices for Dcor compared to Pcor (Fig. 5F). The improvement was substantial for the Power ROIs (d = 2.2, t(213) = 32.2, p < 0.001) and the Craddock ROIs (d = 2.88, t(213) = 42.1, p < 0.001) and even larger for the AAL ROIs (d = 3.65, t(213) = 53.4, p < 0.001). ROIs that were less homogeneous tended to show greater increases in between-participant similarity from Pcor to Dcor for the Craddock (r = 0.59, p < 0.001) and the Power ROIs (r = 0.46, p < 0.001), but not for the AAL ROIs (r = 0.17, p = 0.08). In contrast, when comparing the univariate Dcor to Pcor, between-participant similarity decreased for the Power ROIs (d = − 0.3, t(213) = − 4.4, p < 0.001) and the Craddock ROIs (d = − 0.17, t(213) = 2.6, p = 0.01) and increased for the AAL ROIs (d = 0.57, t(213) = 8.3, p < 0.001). Between-participant similarity was significantly lower for the unsigned Pcor values compared to the original Pcor estimates, bolstering the significance of the improvement with Dcor (Dcor vs. unsigned Pcor; Power ROIs, d = 2.8, t(213) = 41, p < 0.001; Craddock ROIs, d = 3.03, t(213) = 44.4, p < 0.001 and AAL ROIs, d = 3.33, t(213) = 48.7, p < 0.001).

### 3.7 Reliability of individual connections

**Fig. 6:**
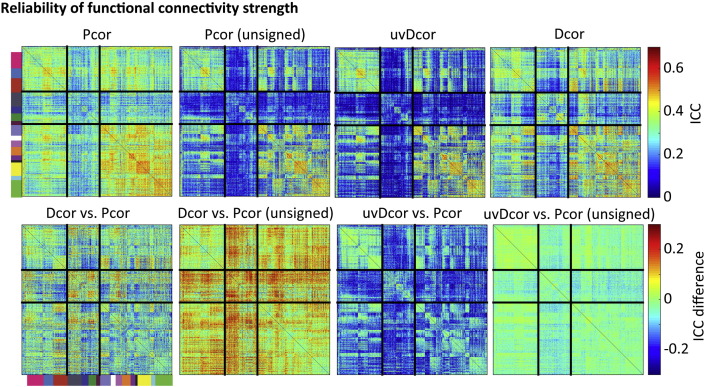
The reliability (ICC) of estimates of functional connectivity strength for each pair of Craddock ROIs.

In Section 3.5, we tested the reliability of the full connectivity matrix; here instead we investigate the reliability of functional connectivity strength for individual connections. These are complementary analyses; while reproducibility of the full connectivity matrix is important for analyses characterizing the entire network (such as graph theory), reliability of single connections are important for research linking connection strength to group membership or behaviour. Using the Craddock ROIs, Fig. 6 shows the average ICC estimates for each of the connection according to each connectivity measure. We found that the reliability was strongly associated with the strength of functional connectivity, with stronger connections being more reliable. The reliability differences between Pcor and Dcor were variable across regions. Subcortical regions tended to show higher reliability for Dcor; connections within cortical networks did not show large differences; while connections between different cortical networks involved in higher cognitive functions tended to show higher reliability for Pcor. This appears to reflect the more restricted range of Dcor, because the reliability improved for almost all connections when comparing Dcor to the unsigned version of Pcor. The univariate Dcor measure also showed poorer reliability than Pcor, which could again be explained by the more limited range of the uvDcor values, since only minimal changes were observed between univariate Dcor and the unsigned Pcor values. Thus it is possible that multivariate Dcor is generally associated with a more reliable estimate of functional connectivity strength, but this obscured by the restricted range of the Dcor measure. Nevertheless, the advantage of Dcor relative to Pcor in Fig. 6 is less prominent than in Fig. 5E and F, suggesting that the primary advantage of Dcor is in reliably measuring the differences between connections, while between-participant differences in the strength of single connections benefit less.

### 3.8 Robustness to the choice of ROIs

**Fig. 7:**
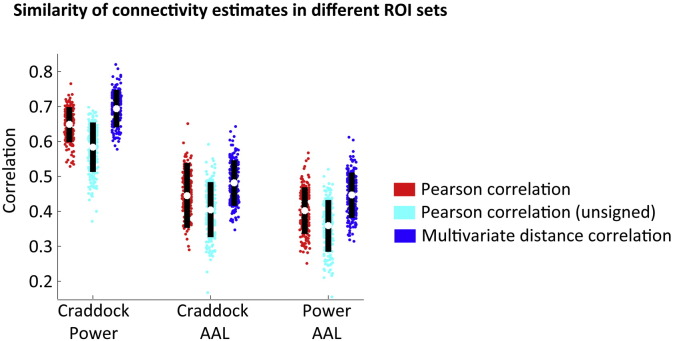
Correlation between voxel-wise connectivity matrices, based on different ROI sets.

A marked advantage of the multivariate approach is that it incorporates information from all the voxels within an ROI, rather than just the most dominant ones. This would suggest that the choice of a specific set of ROIs should have less impact on the observed connectivity patterns than for the traditional univariate approach. Because we cannot compare connectivity matrices directly between different sets of ROIs, we projected the ROI-based connectivity matrices back onto the voxel level. Then we computed the correlation between these voxel-wise connectivity matrices, using only those voxels that were covered by both ROI sets. The results show that the correspondence between different ROI sets was indeed increased for Dcor compared to Pcor for the comparison between Craddock and Power ROIs (d = 1.06, t = 15.6, p < 0.001) the comparison between AAL and Power ROIs (d = 0.89, t = 13, p < 0.001), and the comparison between AAL and Craddock ROIs (d = 0.48, t = 7, p < 0.001; see Fig. 7). Comparisons between Dcor and unsigned Pcor revealed even larger improvements for Dcor in the correspondence between all three ROI sets (Craddock-Power d = 2.35, t = 34.4, p < 0.001; Craddock-AAL d = 1.73, t = 25.3, p < 0.001; AAL-Power d = 1.96, t = 28.7, p < 0.001).

### 3.9 Effects of additional pre-processing

**Fig. 8:**
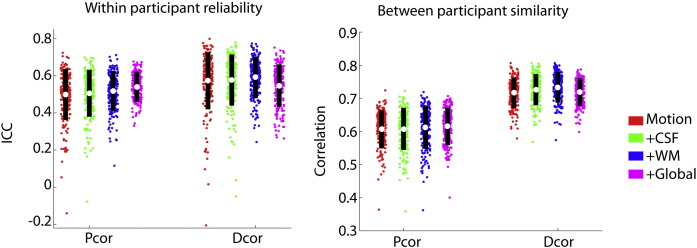
Within-participant reliability and between-participant similarity for different data pre-processing options. White dots indicate the mean, while black bars delineate one standard deviation around the mean.

We examined how the reliability and between-participant similarity of Dcor and Pcor were affected by regressing out nuisance signals, such as the CSF, white matter and global signal (see Fig. 8). We compared four different options: no nuisance signals (except for motion parameters and their derivatives, N), CSF signal regression (C), CSF + WM signal regression (CW), CSF + WM + global signal regression (CWG). For Pcor, reliability was highest for CWG (d = 0.37 relative to N, t(213) = 5.4, p < 0.001; relative to C, d = 0.39, t(213) = 5.7, p < 0.001; relative to CW, d = 0.37, t(213) = 5.4, p < 0.001). Similarly, between-participant similarity was highest for CWG (relative to N, d = 0.27, t(213) = 3.9, p < 0.001; relative to C, d = 0.25, t(213) = 3.7, p < 0.001; relative to CW, d = 0.1, t(213) = 1.5, p = 0.13).

Dcor showed the highest reliability for CW (d = 0.17 relative to N, t(213) = 2.4, p = 0.016). Adding the global signal, or removing the WM signal, was associated with lower reliability relative to CW (d = − 0.71, t(213) = − 10.4, p < 0.001, d = − 0.2, t(213) = − 2.9, p = 0.004; respectively). Also, the similarity between participants was highest for CW (relative to N, d = 0.63, t(213) = 9.3, p < 0.001; relative to C, d = 0.43, t(213) = 6.2, p < 0.001; relative to CWG, d = 0.62, t(213) = 9.1, p < 0.001).

### 3.10 Effects of participant motion

We examined how participant motion affected the different functional connectivity estimates. To this end, we correlated the connectivity estimates with the total amount of motion (see Section 2.4) across participants, for each connection. Two outcome measures that are typically used are the average correlation between connectivity and motion (across distances) and the distance-dependence of these correlations (given that the effects of motion on connectivity estimates are stronger for pairs of ROIs that are close together; Patel et al., 2014, Power et al., 2015). While the average correlation with motion was low for all measures (Dcor: r = 0.12, Pcor, r = − 0.01, unsigned Pcor, r = 0.002), the distance dependence was stronger for Pcor and unsigned Pcor than for Dcor (r = − 0.25, r = − 0.23, and r = − 0.14, respectively).

**Fig. 9:**
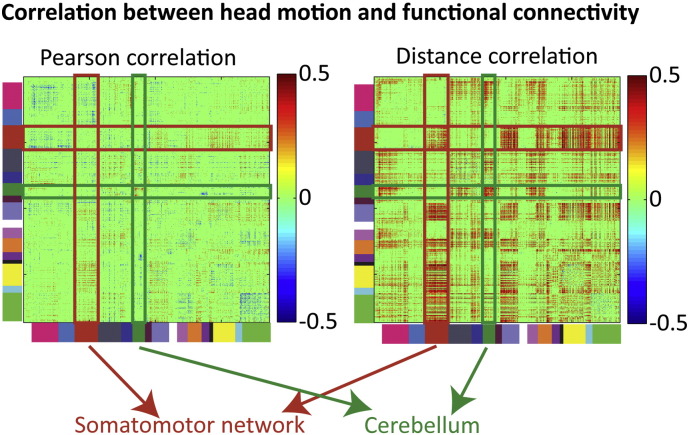
Significant correlations (p < 0.001) between functional connectivity and head motion for Dcor and Pcor, in the Craddock ROI set. ROIs are ordered by functional network, as indicated by the colors on the left side and bottom of the functional connectivity matrices. The red bars indicate regions associated with the somatomotor network, green bars indicate regions in the cerebellar network.

Fig. 9 shows how average head motion was associated with functional connectivity, for both Dcor and Pcor (the association is very similar for unsigned Pcor). It is noteworthy that for Dcor, but not Pcor, the associations with increased motion included greater connectivity between the motor cortex and higher order functional networks, as well as greater connectivity within the cerebellum and between the cerebellum and the visual network. Given the localized nature of these motion effects, and the fact that they do not appear to be strongly distance dependent, it may be that some of the motion-related connectivity differences observed with Dcor reflect true functional connectivity differences related to head motion, rather than motion artefacts (Zeng et al., 2014).

### 3.11 Structural connectivity

**Fig. 10:**
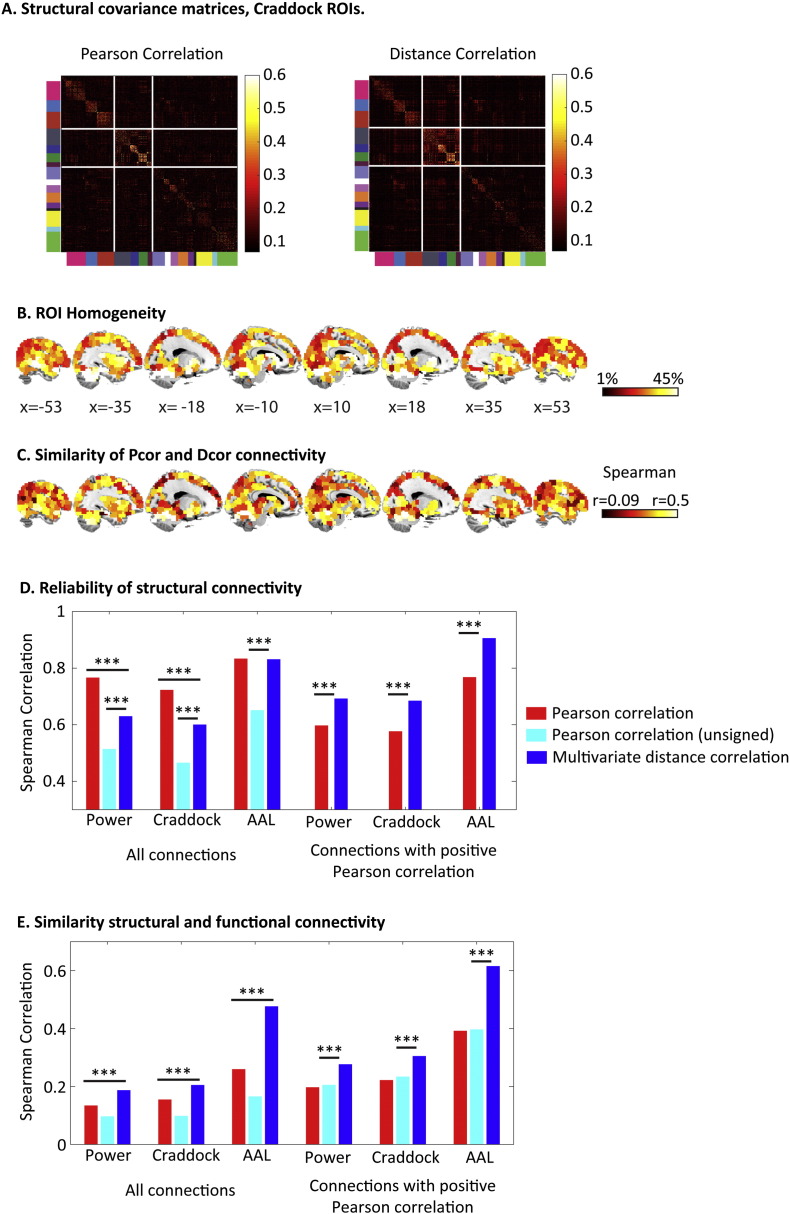
**A**. Matrices of functional connectivity and structural connectivity for the Craddock ROIs, using Pcor or Dcor. Unsigned Pcor values were used to emphasize differences due to the multivariate method, rather than the absence of negative correlations. ROIs are ordered by the functional networks depicted in Fig. 1, as indicated by the colors on the left side and bottom of the functional connectivity matrices. **B**: Regional differences in ROI homogeneity of grey matter volumes. **C**. Correlation between Pcor and Dcor structural connectivity patterns from each brain region to all other regions. Unsigned Pcor values were used to quantify the similarity. **D**. Reliability of the structural connectivity matrix (similarity between session 1 and session 2). **E**. The similarity between structural connectivity and functional connectivity for each of the ROI sets. *** p < 0.001.

Fig. 10 shows the structural connectivity matrices for Pcor and Dcor for the Craddock ROIs. The distribution of connection strengths is clearly positively skewed (which is why we switched to Spearman's correlation for analyses below). Nonetheless, the matrices show a clear correspondence to the functional connectivity matrices in Fig. 4. The same network structure is apparent for both Dcor and Pcor, with strong within-network connectivity and weak between-network connections (see Fig. 10A). Parietal and frontal regions in particular showed prominent changes in structural connectivity patterns when comparing Dcor to Pcor (see Fig. 10C).

First, we investigated ROI homogeneity for the grey matter data. This is shown for the Craddock ROIs in Fig. 10B (since these have equally sized ROIs that cover the whole brain). Similar to functional connectivity, we found that ROIs with high homogeneity showed more similar structural connectivity patterns for Dcor and Pcor (Power r = 0.66, p < 0.001; Craddock r = 0.7, p < 0.001; AAL r = 0.75, p < 0.001). The percentage of variance that could be explained by the first principal component was around 33% for the Power ROIs, 31% for the Craddock ROIs and 31% for the AAL ROIs. Notably, we observed that subcortical regions showed particularly high homogeneity for grey matter volumes, while these regions showed the lowest homogeneity for the fMRI data.

We also looked at the reliability of the structural connectivity patterns, by correlating the association matrices from session 1 with the matrices from session 2 (see Fig. 10D). The significance of the differences between these correlations for Pcor and Dcor was based on permutation testing. For the Power and Craddock ROIs, the reliability was significantly higher for Pcor than Dcor (both p < 0.001), while no significant difference was observed for the AAL ROIs (p = 0.48). However, for all three ROI sets, we found that Dcor was significantly more reliable than the unsigned Pcor measure (all p < 0.001). These results suggest that the lower reliability of Dcor compared to Pcor is due to the wider range of the Pcor measure. To explore this further, we investigated the reliability of only those connections that had a positive Pcor value in both sessions. Here we indeed found that Dcor connectivity values were significantly more reliable than Pcor values (all ROI sets p < 0.001).

For the Craddock and Power ROIs, a substantial number of connections were set to zero for Dcor, as they had negative estimates of distance covariance (and therefore had a non-significant association). Therefore, we performed an additional check in which the same number of connections that were zero for Dcor were also set to zero for Pcor (replacing the weakest connectivity estimates) and we recomputed the reliability for unsigned Pcor values. In this analysis the differences between Pcor and Dcor remained highly significant (p < 0.001) suggesting that observed differences are not due to this difference in the distribution of connectivity estimates.

Finally, we related functional connectivity matrices (averaged across participants) to structural connectivity patterns (see Fig. 10E). Although the precise relationship between structural and functional connectivity is not yet known, there is growing evidence that regions with strong structural connectivity also show strong functional connectivity (see e.g. Alexander-Bloch et al., 2013, Hermundstad et al., 2013, Honey et al., 2009). To relate structural and functional connectivity, we first used a naïve approach in which we simply correlated the two matrices. Here we observed significantly higher similarity between structure and function for Dcor relative to Pcor or unsigned Pcor (all ROI sets p < 0.001). Because it is not clear from the previous literature how negative structural connections would be related to functional connectivity, we performed additional analyses in which we took only positive structural and functional connections into account. As expected, this analysis resulted in a better correspondence between function and structure. For all ROI sets, Dcor resulted in higher similarity between structure and function than Pcor and unsigned Pcor (all p < 0.001).

## 4 Discussion

Here we conducted an extensive investigation of distance correlation as an alternative to Pearson correlation for ROI-based analysis of functional connectivity, using both simulated and real data. Distance correlation is different from Pearson correlation in two important ways: it is able to detect non-linear associations between brain regions and it can use multivariate patterns to measure the dependence between two ROIs, without a need for signal averaging. Our simulations confirmed situations where distance correlation detects connectivity that is missed by conventional Pearson correlations. Our results on real data demonstrated that distance correlation tends to result in improved estimates of functional connectivity; as demonstrated by increased reliability, higher between-participant similarity, better robustness to the choice of ROI sets and higher similarity between functional and structural connectivity.

To pinpoint which aspect of distance correlation caused this improvement, we additionally investigated a univariate version of distance correlation, which was based on the average signal within each ROI (similar to Pearson). We found that this univariate measure resulted in slightly lower estimates of reliability and between-participant similarity than the Pearson correlation, suggesting that it is not the non-linearity, but rather the multivariate nature of distance correlation that is associated with better connectivity estimates. This is in line with a previous study, which has demonstrated that in some cases, multivariate connectivity methods can detect connections which cannot be observed in standard univariate analyses (Anzellotti et al., 2016). It is also in line with other work showing that non-linear interactions represent only a small proportion of the regional interactions found with fMRI (Hlinka et al., 2011). Nevertheless, it is also possible that on the scale of single voxels, there are non-linear interactions that contribute significantly to the functional interactions that are measured with multivariate distance correlation, but which cannot be detected after averaging the signals within each ROI.

Differences in functional and structural connectivity estimates between distance correlation and Pearson correlation were most prominent for ROIs with low homogeneity; these were regions in which the connectivity patterns were not well captured by a single principal component. Subcortical regions especially showed relatively low homogeneity of functional connectivity, whereas sensory and motor region were highly homogenous. These results suggest that subcortical connectivity patterns may have been misrepresented in previous ROI-based functional connectivity studies using Pearson correlations. In contrast, in the analysis of structural connectivity, we observed that subcortical regions showed relatively high homogeneity in their connectivity patterns across participants, while frontal and parietal regions showed relatively low homogeneity. This may be associated with the cytoarchitectonic properties of these regions: Different cortical layers may contribute differentially to each voxel's estimate of grey matter volume, and some voxels may overlap with the grey-white matter boundary, resulting in lower homogeneity for structural connectivity in cortical versus subcortical regions. In contrast, for functional connectivity, it is known that small subcortical nuclei are involved in cortico-subcortical loops with different cortical regions, which may be one of the reasons for low subcortical homogeneity.

Although distance correlation exhibited greater consistency within and across participants in our data, this does not mean that it is more accurate (i.e., closer to the true connectivity), since a metric can be consistently biased. In other words, it remains possible that some other nuisance signal affected the data in the same way for different scans and different participants, to which the distance correlation happened to be particularly sensitive, and this lead to the improvement in reliability and between-participant similarities. This was one reason for comparing connectivity patterns across functional and structural measures. Previous research suggests that regions that have strong structural connections also tend to show strong functional connectivity (Alexander-Bloch et al., 2013, Hermundstad et al., 2013, Honey et al., 2009). Since structural and functional connectivity are derived from separate data, there are few sources of bias that are common to both structural and functional connectivity. In fact, we could think of only two potential sources of bias that could artificially increase the correspondence between structural and functional connectivity in Dcor versus Pcor: the number of voxels in an ROI and regional differences in the amount of noise. Our simulations showed that Dcor is not affected by the number of voxels in an ROI and that the relative levels of noise in each ROI affect Dcor and Pcor in a similar way. Moreover, our analyses showed that voxel-specific noise did not have a significant impact on our results. So even though perfect correspondence between structural and functional connectivity would never be expected, since these represent two distinct aspects of the connectome, our observation of better correspondence between structural and functional connectivity is important, in arguing against a consistent bias in Dcor being the cause of its increased reliability and between-participant similarity.

The improved function–structure correspondence therefore suggests that distance correlation estimates of connectivity may be a closer approximation of the underlying true connectivity. This was further supported by analyses of robustness to the choice of ROIs: We found that connectivity in different ROI sets was more similar when connectivity estimates were based on distance correlation, than when based on Pearson correlation. This suggests that the use of a multivariate measure preserves more information about the connectivity patterns of each of the voxels within an ROI. Note that this does not mean that the choice of ROI-set will no longer affect the outcomes of a study once distance correlation is used; choosing an ROI set that more closely corresponds to the brain's functional architecture will always result in more valid inferences about functional connectivity.

We also examined how reliability and between-participant similarity were affected by pre-processing choices. We also observed that for distance correlation, regression of CSF and white matter signals led to higher reliability and increased between-participant similarities, while global signal regression resulted in lower reliability. Given that the use of global signal regression is already widely debated, because it can contain neural signal in addition to noise (Murphy et al., 2009, Saad et al., 2012, Schölvinck et al., 2010), these results suggest that global signal regression is not advisable for functional connectivity analyses using distance correlation.

One potential limitation of the distance correlation measure is that it is not able to distinguish negative and positive correlations. Negative correlations in functional connectivity have been observed between functionally distinct networks, and occur most prominently after global signal regression (Fox et al., 2005, Fransson, 2005). They can also be observed when global regression is not applied, though they are generally weak and less reliable than positive correlations (Chang and Glover, 2009, Schwarz and McGonigle, 2011, Shehzad et al., 2009), and their neurophysiological basis is still unclear (Fox et al., 2009, Murphy et al., 2009).

One implication of distance correlation being positive is that its reduced range (0–1) will potentially decrease its reliability. That is why we also compared distance correlation to an unsigned Pearson correlation, where we flipped the sign of negative Pearson values, leading to the same range of 0–1. In no case did unsigned Pearson perform better than distance correlation. Indeed, unsigned Pearson correlation performed worse than signed Pearson correlation, as would be expected statistically from its reduced range. Only in two cases did signed Pearson correlation perform better than distance correlation: in terms of reliability of individual functional connections, and in terms of overall reliability of structural covariance. It is plausible that these cases are a statistical consequence of the greater range of (signed) Pearson correlation; however, it is additionally possible that negative connections are an important feature of connectivity.

Another potential limitation, mentioned above, is that distance correlation can be biased when the data possess high autocorrelation. In this case, connectivity estimates around zero become inflated. This restricts the use of this method to data that have been pre-whitened. Indeed, our results show that pre-whitening the data (after high-pass filtering) leads to more reliable results, for both Pearson and distance correlation. In exploring this issue, we also discovered that band-pass filtering of the data produces a high temporal autocorrelation that cannot be removed by conventional approaches to pre-whitening fMRI data. This cautions against the use of band-pass filtering in conjunction with correlational measures of functional connectivity; a problem that has already been noted for Pearson correlation (Arbabshirani et al., 2014). Band-pass filtering has traditionally been justified on the basis that high frequency components of the BOLD signal are likely to reflect noise. However, this has recently been called into question by a number of studies demonstrating that high frequency signals also contribute to functional connectivity (Boubela et al., 2013, Chen and Glover, 2015, Gohel and Biswal, 2014, Kalcher et al., 2014) and that high-pass filtering (compared to band-pass filtering) is associated with better signal-noise separation, test–retest reliability and group discriminability (Shirer et al., 2015).

To conclude, these results suggest that using multivariate distance correlation is an important step forward in obtaining reliable and robust estimates of functional and structural connectivity between discrete regions of interest. Using an average signal to measure connectivity between two ROIs can result in a significant bias of connectivity estimates, and may exacerbate differences between participants that owe to anatomical differences, rather than connectivity differences. This may be especially important when comparing individuals with known differences in anatomy, such as patients with neurodegenerative diseases, or older and younger participants. Distance correlation is easy to implement and fast to compute, which makes it especially suitable for whole brain ROI-based connectome analyses.

## Software note

Matlab scripts for computing the double-centred and the U-centred estimates of Dcor are available from http://imaging.mrc-cbu.cam.ac.uk/imaging/Geerligs_DistCor. We also provide an example script for functional connectivity analyses, which includes the extraction of data from ROIs, pre-whitening and nuisance regression.

## Figures and Tables

**Fig. 1:**
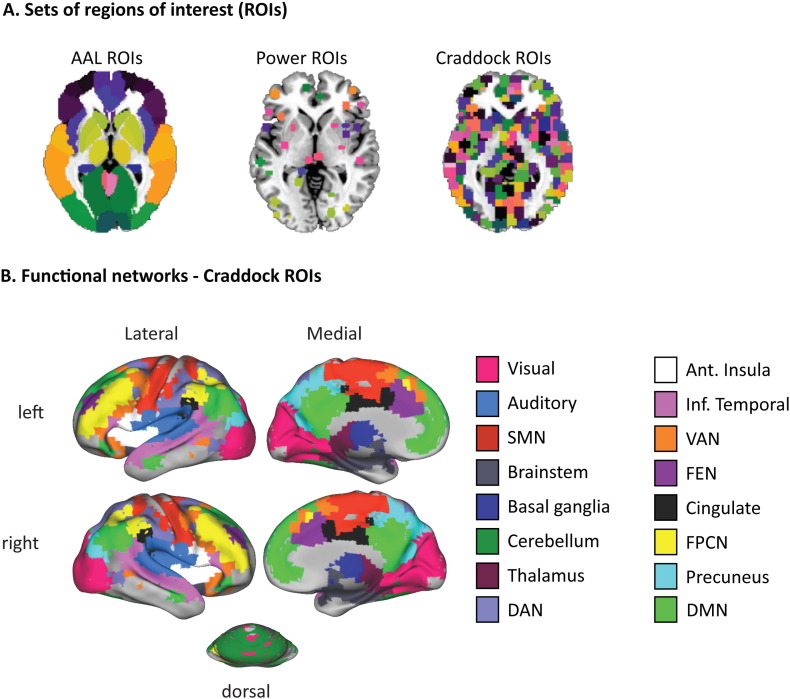
**A**: Illustration of the ROIs in each set. Craddock ROIs contain 23 voxels on average, while Power ROIs contain 65 voxels on average. For the AAL atlas, ROIs typically contain thousands or even tens of thousands of voxels. **B**: Functional networks based on the Craddock ROIs, defined in Geerligs et al. (2015) from a superset of participants based on Pearson correlations. These networks are used to order the functional connectivity matrices in Fig. 4, Fig. 6, Fig. 9, Fig. 10.
